# MEL-IA: An Interoperable AI System for Multimodal Skin Lesion Classification in Hospital Settings

**DOI:** 10.1007/s10916-026-02424-y

**Published:** 2026-06-12

**Authors:** Pablo Candela Córcoles, Alberto De Ramón Fernández, Marcelo Saval Calvo, Jose María Salinas Serrano, Diego Guijarro Peral, Daniel Ruiz Fernández

**Affiliations:** 1 Bioinspired Engineering and Health Informatics research group (IBIS), University of Alicante, Alicante, Spain; 2 Department of Computer Technology (DTIC), University of Alicante, Alicante, Spain; 3 Department of Health Informatics, San Juan Hospital, Alicante, Spain

**Keywords:** Skin Lesion Classification, Interoperability, Mobile Health (mHealth), Hospital Information Systems

## Abstract

Skin cancer represents a growing healthcare challenge, motivating the development of AI sytems that can support lesion assessment while integrating efficiently into clinical workflows. This study presents MEL-IA (MobilE skin Lesion dIAgnosis), an interoperable AI system designed for automated skin lesion classification and full integration with hospital information systems. Methods: The system combines dermatoscopic images and structured clinical metadata using an EfficientNet-B4 - based multimodal model trained on the ISIC 2019 BCN_20000 and MSK subsets. Internal robustness was assessed through stratified 5-fold cross-validation, and external generalization was evaluated on the independent HAM10000 dataset. Interoperability and operational performance were validated through deployment in a real hospital environment using HL7, DICOM, PACS, and HIS/RIS systems. Results: We evaluated internal robustness using stratified five‑fold cross‑validation, with global accuracy of 0.86, macro F1-score of 0.85, and AUC values above 0.97. The external evaluation showed that the model generalizes well under different acquisition conditions, achieving an accuracy of 0.84 and balanced accuracy of 0.65, with sensitivities of 0.60 for melanoma 0.72 for basal cell carcinoma. The multimodal model outperformed the image model baseline across all classes. Technical deployment confirmed full interoperability, > 99% successful study integration, and near real time processing. Conclusions: MEL-IA provides multimodal skin lesion classification capabilities and integrates smoothly into hospital infrastructures based on standards. The results demonstrate technical feasibility, interoperability, and operational viability for deployment within dermatology oriented clinical workflows. Further clinical validation studies are warranted to assess the system’s impact on diagnostic decision-making and patient outcomes.

**Supplementary Information:**

The online version contains supplementary material available at 10.1007/s10916-026-02424-y.

## Highlights

MEL-IA integrates AI-based skin lesion classification with full interoperability in hospital systems.

The EfficientNetB4-based multimodal model achieved consistent accuracy (≈ 0.86) and AUC > 0.97.

A mobile application enables structured image acquisition, metadata management, and real time diagnosis.

Deployment in a real hospital environment confirmed technical feasibility.

**Supplementary Information:**

The online version contains supplementary material available at 10.1007/s10916-026-02424-y.

## Introduction

Skin cancer is an urgent global health issue, with particularly high prevalence in Europe, where about 144 000 new melanoma cases were reported in 2020 [1]. In the United States, it is the most common cancer, affecting one in five people, causing over 5 million non-melanoma and more than 100 000 melanoma cases annually. Worldwide, dermatologic conditions affect nearly 1.8 billion people [2]. Early detection remains critical for improving clinical outcomes and reducing treatment complexity.

Despite preventive initiatives, the diagnostic process for skin lesions remains complex and time-consuming, heavily dependent on dermatologist expertise. Conventional diagnostic methods, although effective, show a high degree of subjectivity and inter-observer variability [3–6], adding to clinician workload.

Artificial intelligence (AI) offers a has the potential for improving dermatologic diagnostics [7–9]. Recent developments in imaging and machine learning (ML) have produced automated systems capable of detecting and classifying lesions with high accuracy. Convolutional neural networks (CNNs), segmentation and classification algorithms, and generative models such as GANs and variational auto-encoders which have achieved strong performance [10–12].

However, bringing these systems into clinical practice remains difficult, especially due to interoperability limitations. Existing apps such as Legit.Health, Skinive, Medgic, and MoleScope [13–16] perform image analysis, although their integration with hospital information system remains limited.

We propose MEL IA (MobilE skin Lesion dIAgnosis), a multimodal AI system that combines lesion classification, mobile image acquisition, and interoperability with hospital information systems. *MEL-IA* classifies skin lesions using dermatoscopic images and structured clinical metadata captured on mobile devices and, by adhering to HL7 and DICOM standards, integrates with existing hospital infrastructures such as PACS and EHR systems, ensuring full data traceability and supporting streamlined specialist referrals. The core contribution of the system lies not in introducing a new CNN architecture, but in the integration of an established multimodal classification model within a hospital environment that supports image acquisition, inference, storage, and clinical data exchange using interoperability standards.

We hypothesize that a multimodal model trained on ISIC 2019 (BCN_20000 and MSK datasets) [17, 18] can maintain consistent classification performance on an independent external dataset (HAM10000) [19] and that embedding the model within an architecture based on interoperability standards supports workflow integration from mobile image capture to PACS/EHR storage within routine dermatology workflows. To address this hypothesis, we investigate three scientific questions: (1) whether a multimodal classifier trained on ISIC 2019 generalizes to an external dataset acquired under different conditions; (2) whether an AI system can be integrated into a real hospital environment using interoperability standards; and (3) whether the combination of mobile acquisition, backend inference, and PACS/EHR integration supports clinical workflows in real time.

## Methods

### Study Design and Overview

The proposed approach focuses on developing a holistic system for skin lesion diagnosis that, by means of ML, classifies using images from mobile devices and integrates the information with the hospital ecosystem. Dermatoscopic image acquisition and analysis are combined with an interoperability framework based on the Mirth Connect platform [20], which enables the structured and secure exchange of clinical data - including HL7 reports, DICOM images, and diagnostic outputs.

**Fig. 1:**
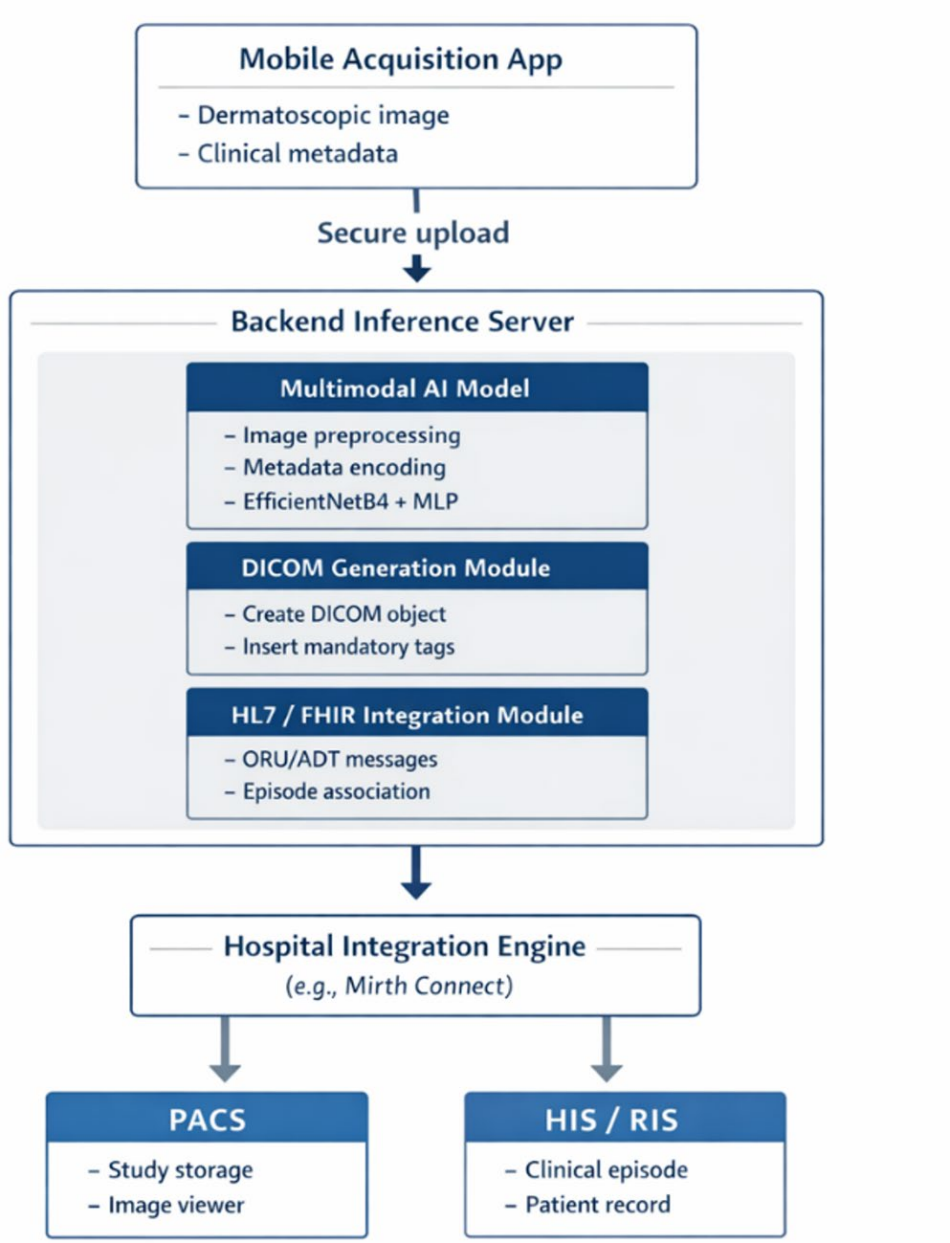
Overview of the MEL-IA system architecture

Figure 1 illustrates the overall workflow of MEL-IA, which includes: (1) mobile acquisition of dermatoscopic images and structured clinical metadata; (2) secure transmission to the backend server; (3) multimodal inference combining image and metadata; (4) generation of DICOM objects and HL7/FHIR messages; and (5) integration with the hospital PACS and HIS/RIS systems through the institutional interoperability engine. This modular design enables independent updates of the AI model while preserving compatibility with existing clinical workflows.

### Dataset Description and Preprocessing

To train and validate the AI model, we selected the BCN_20000 and MSK subsets[17, 18] of the ISIC 2019 Challenge dataset, a widely recognized benchmark comprising 25,331 dermatoscopic images across eight diagnostic categories. These two components were chosen because they provide the largest volume of images and the most complete structured metadata (age, sex, anatomical site), enabling robust multimodal training.

HAM10000 was reserved exclusively for external validation [19]. Despite its high quality, HAM10000 contains fewer samples, exhibits different acquisition characteristics, and includes more limited metadata fields, making it less suitable as a primary source for multimodal learning. This design prevents dataset leakage between training and evaluation splits and ensures that the final multimodal model is assessed on an independent dataset acquired under different clinical and technical conditions.

Three classes - Dermatofibroma (DF), Vascular Lesions (VASC), and Squamous Cell Carcinoma (SCC) - were excluded due to extremely low representation (239, 253, and 628 cases, respectively) and their absence in HAM10000, as their inclusion would have led to unstable gradients and unreliable calibration.

Metadata preprocessing involved label-encoding categorical variables and imputing missing values using both KNNImputer and HotDeck, ensuring consistency across samples. All images were normalized using ImageNet statistics with mean values of 0.485, 0.456, and 0.406, and standard deviations of 0.229, 0.224, and 0.225 to maintain compatibility with the pretrained EfficientNetB4 backbone and avoid distributional mismatch.

**Fig. 2:**
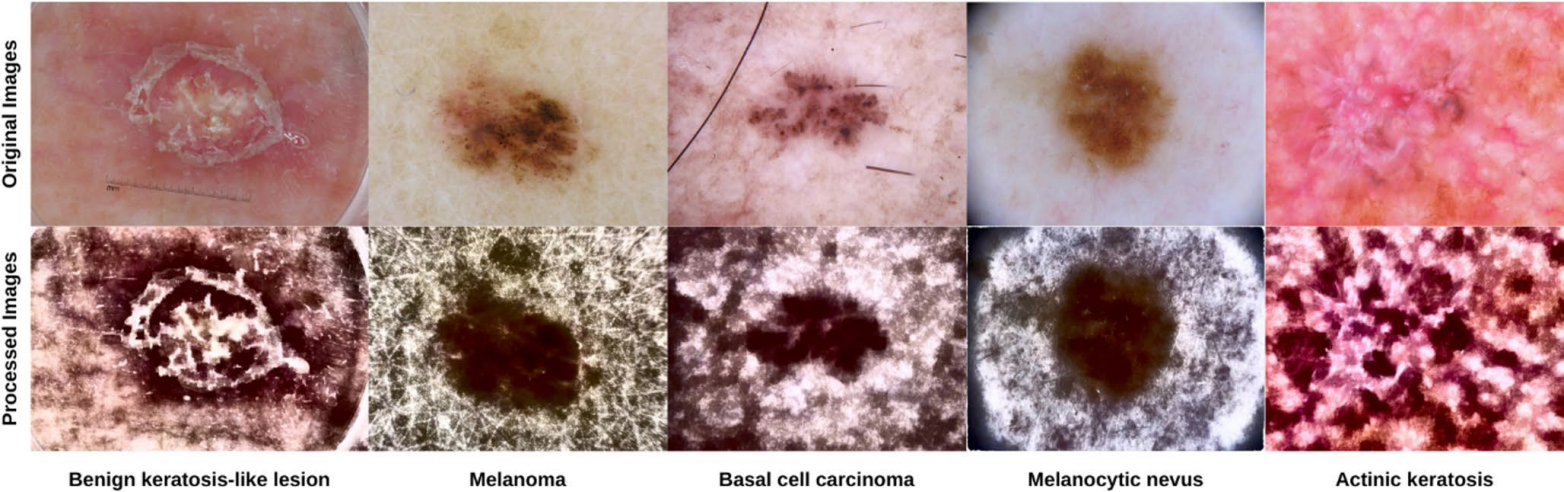
Visual comparison of original and preprocessed images for five lesion categories

Dermatoscopic image preprocessing aimed to enhance visual quality by emphasizing relevant structures and minimizing artifacts (Fig. 2). Hair removal was performed using the DullRazor algorithm [21], while local contrast enhancement was applied using CLAHE [22]. Additional transformations including resizing, gamma correction, unsharp masking, and edge detection via Canny [23] and Hough Transform [24] were applied to optimize lesion visibility and support downstream segmentation. Pretrained models from Torchvision’s segmentation suite were used to refine lesion boundaries.

To obtain a balanced multimodal training set and prevent the model from being biased toward the majority class, we applied targeted oversampling to the underrepresented categories using standard geometric and photometric augmentations (translation, rotation, cropping, contrast adjustments). No synthetic images were generated. Each minority class was augmented until matching the size of the largest class, resulting in a balanced dataset of 15,000 samples. The final class distribution after preprocessing and augmentation is shown in Table 1.

**Table 1:** Final class distribution after preprocessing and targeted data augmentation

Label	Lesion Type	Original Samples in ISIC2109	Samples selected [17, 18]	Total after DA
**MEL**	Melanoma	4,522	3,000	3,000
**NV**	Nevus	12,875	3,000	3,000
**BCC**	Basal Cell Carcinoma	3,323	2,719	3,000
**AK**	Acnitic Keratosis	867	540	3,000
**BKL**	Benign Keratosis	2,624	1,535	3,000

### Implementation Pipeline

**Fig. 3:**
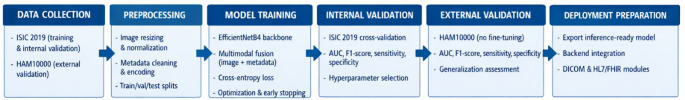
Complete implementation pipeline of MEL-IA

Figure 3 summarizes the implementation pipeline of MEL-IA, covering the full process from data preprocessing and multimodal model training on ISIC 2019 to internal validation, external validation on HAM10000, and final deployment within the hospital environment. This pipeline highlights the separation between the experimental evaluation of the AI model and its clinical integration and distinguishing the research workflow from the operational system architecture described in the following section.

### System Architecture

From an architectural perspective, the system has been designed as a distributed, flexible, and scalable platform capable of adapting to diverse hospital environments. To support this adaptability, the use of Service-Oriented Architecture (SOA) has been chosen, leveraging RESTful web services [25] to ensure low coupling between components and a strong emphasis on clinical data exchange. This design facilitates integration with heterogeneous databases and hospital information systems, promoting interoperability across varied infrastructures.

**Fig. 4:**
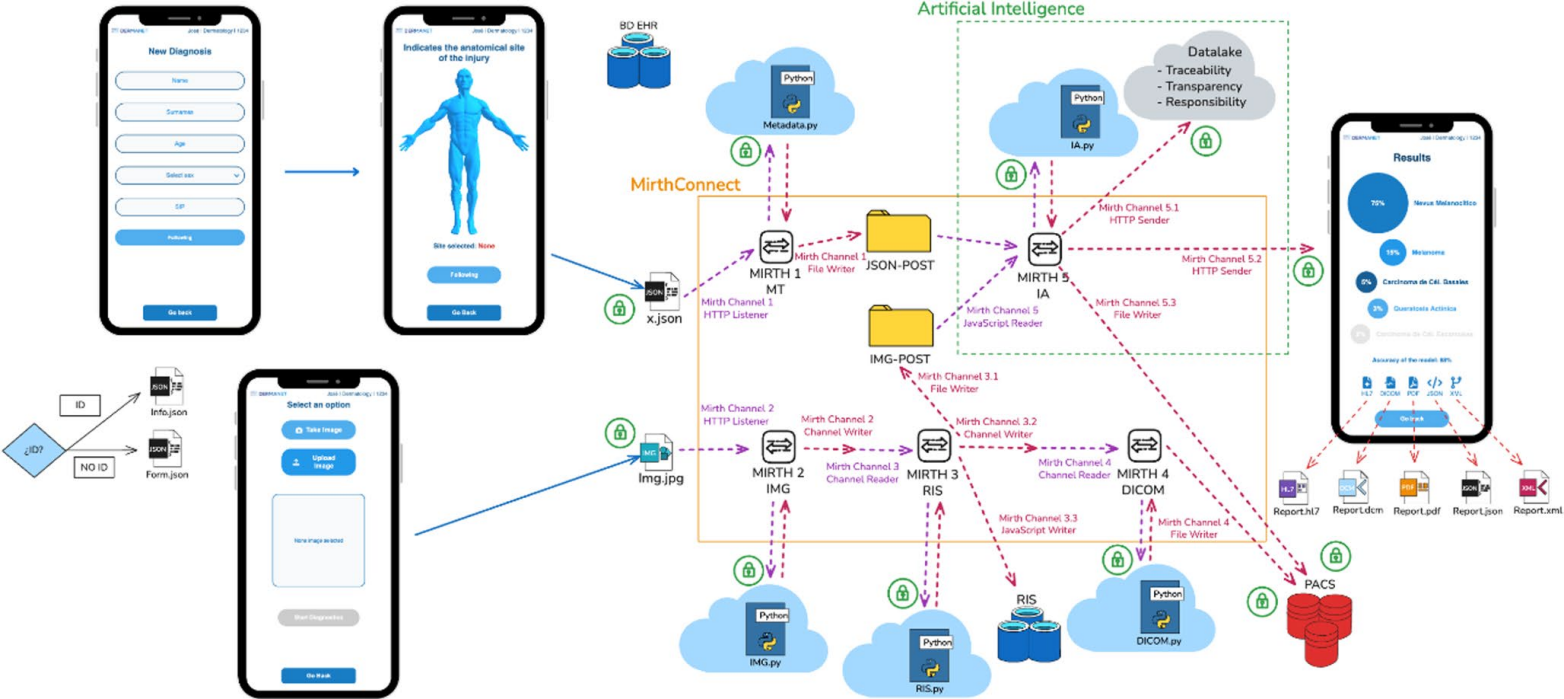
Conceptual workflow of MEL-IA architecture

The distributed nature of the system not only guarantees compatibility with existing clinical workflows but also establishes a foundation for future modular expansion into other medical specialties. The overall workflow of the proposed system is illustrated in Fig. 4, which identifies three essential building blocks that constitute the core foundation of the project. These blocks collectively support its clinical functionality and interoperability.

First, a mobile application has been developed to serve both healthcare professionals and patients. This application is designed to enable the structured acquisition of dermatoscopic images, associate relevant clinical metadata, manage patient medical histories, and display diagnostic outcomes generated by the AI module. As the primary interface of the platform, it facilitates secure and efficient user interaction, ensuring accessibility and usability across diverse clinical settings.

Second, a multimodal and multiclass classification algorithm was implemented, specifically trained for the automated analysis of dermatoscopic images. This ML model can categorize skin lesions based on their morphological and clinical characteristics, thereby supporting lesion classification and reducing the time required to obtain automated predictions.

Finally, a centralized integration module has been developed to act as the system orchestrator, coordinating communication among components through the Mirth Connect integration engine^1^[20]. This open-source platform is well-suited for clinical environments, as it supports standardized message formats such as HL7, XML, and JSON. By configuring dedicated channels, Mirth Connect enables the automated reception, transformation, and distribution of data across heterogeneous systems, applying specific rules for filtering, mapping, and routing. This architectural approach ensures interoperability with existing hospital infrastructures, EHR systems and PACS.

Table 2 provides a summary of the integration channels implemented and outlines their respective roles within the data exchange workflow shown in Fig. 4.

**Table 2:** Summary of Implemented Data Exchange Channels for EHR and PACS Integration

Channel	Source Connector	Destination Connector	Details	Description
**IMG**	HTTP Listener	Channel Writer (RIS)	Executes a JavaScript script that calls IMG.py to process images using a series of filters.	Channel dedicated to initial image processing.
**MT**	HTTP Listener	File Writer (METADATA)	Calls MT.py and saves the output as JSON in a special folder that will be used later.	Channel for diagnostics and metadata extraction.
**DICOM**	Channel Reader	File Writer (PACS PROCESSED) File Writer (PACS ORIGINAL)	Executes DICOM.py to process DICOM images, storing both the processed and original images in the PACS.	Channel for storing DICOM images in PACS.
**RIS**	Channel Reader	File Writer (IMG-POST) JavaScript Writer (APPOINTMENT) Channel Writer (DICOM)	Calls RIS.py to process RIS appointment data, manages image storage in a separate folder along with metadata, and integrates appointment info into the database.	Channel that integrates RIS information and manages appointments.
**AI**	JavaScript Reader	HTTP Sender (APP) File Writer (PACS)	Every 3 s, the input connector checks for matching files in the metadata and image folders. Both must share the same server-generated ID. When a match is found, the AI classifier is executed, and the prediction is sent via an HTTP POST request in application/json format.	AI-based classification channel.

The three core components of the system - the mobile application, the ML classifier, and the integration module - were designed following the SOA principles of low coupling and high cohesion. This architectural strategy facilitates modular code reuse, simplifies system maintenance (including error detection and resolution), and optimizes both diagnostic workflows and patient follow-up. Consequently, it supports a more connected, efficient, and interoperable healthcare delivery model.

#### Data workflow

The data flow begins with the acquisition of a dermatoscopic image and the entry of a minimum of clinical information (patient age, sex, and anatomical location of the lesion) via a structured input form. These data are transmitted to the system through secure (see Fig. 4), independent HTTP POST requests over HTTPS one for the clinical metadata and another for the captured image.

Upon reception, the metadata is processed by a dedicated channel on the Mirth Connect platform, referred to as MT in Figure. 4. This channel is responsible for transforming and mapping the data into the format required by the ML model. In parallel, the preprocessed image is transmitted through an independent channel named IMG, where it is temporarily stored until it can be paired with the corresponding metadata for joint analysis.

Simultaneously, the image follows a secondary integration path via the RIS and DICOM channels, enabling its incorporation into the hospital’s PACS. This ensures complete traceability of the image as part of the patient’s clinical study.

Once both the image and metadata are available, the system processes them together using the multiclass classifier (AI classification in Fig. 4). The resulting diagnosis can be exported in multiple formats, including those compatible with clinical standards such as HL7 and DICOM.

The final output is stored as a new series associated with the original study in the PACS, consolidating both the image and the diagnostic result into a single structured clinical record.

Table 3 summarizes the system’s core requirements. Functional requirements (RF) include dermatological image management, ML based skin lesion analysis, medical staff access control, reporting, and integration with clinical environments. These ensure that the system performs essential tasks related to diagnosis and workflow optimization.

Non-functional requirements (RnF) address quality attributes such as security, scalability, usability, and fault tolerance, which are critical for ensuring system reliability and performance. Together, these requirements define a robust and adaptable healthcare solution.

**Table 3:** Functional and Non-functional requirements

Identifier	Functionality Name	Sub-identifier	Sub-functionality Name	Identifier	Functionality Name
RF.1	Dermatological Image Management	RF.1.1	Manual Capture	RnF1	Security
		RF.1.2	Image Upload	RnF2	Scalability
		RF.1.3	Image Preprocessing	RnF3	Usability
RF.2	Skin Lesion Analysis Using AI	RF.2.1	Analysis in real time	RnF4	Compatibility
		RF.2.2	Early Detection	RnF5	Performance
RF.3	Analysis History	RF.3.1	History Management	RnF6	Maintainability
		RF.3.2	Image Comparison	RnF7	Availability
RF.4	Medical Staff Management	RF.4.1	Profile Creation and Validation	RnF8	Fault Tolerance
		RF.4.2	Healthcare Professional Access	RnF9	Portability
		RF.4.3	Access Authorization	RnF10	Connectivity
RF.5	Reporting and Data Export	RF.5.1	Visual and Numerical Reports	RnF11	Multi-user Management
		RF.5.2	Results Export		
RF.6	Integration with Clinical Environments	RF.6.1	Connection with Medical Databases		
		RF.6.2	Report Delivery to Clinics		
RF.7	AI Algorithm Customization	RF.7.1	Personalized Training		
		RF.7.2	Continuous Improvement		

### Artificial Intelligence Model

Current literature about image classification systems are dominated by Deep Learning approaches, which consistently achieve the highest performance in medical imaging tasks. Although many architectures have been proposed over the past decade, our deployment scenario imposes strict requirements on both portability and computational efficiency. For this reason, EfficientNet‑B4 was selected as the base model due to its strong performance in dermatology classification and its favorable accuracy‑to‑efficiency ratio [26].

EfficientNet employs compound scaling of depth, width, and resolution, enabling it to achieve an optimal balance between accuracy and computational cost. Compared to earlier architectures such as MobileNet [27], ResNet [28], and Inception [29], EfficientNet‑B4 [30] has shown superior generalization capabilities - an essential property for dermatoscopic images, which require the detection of subtle and heterogeneous visual patterns. EfficientNet architectures have also consistently ranked among the top performers in ISIC challenges and dermatology benchmarks, outperforming lightweight models such as MobileNet V2/V3 in diagnostic accuracy while requiring fewer parameters and lower inference time than deeper CNNs including ResNet‑50/101 [31] or DenseNet‑201 [32].

**Fig. 5:**
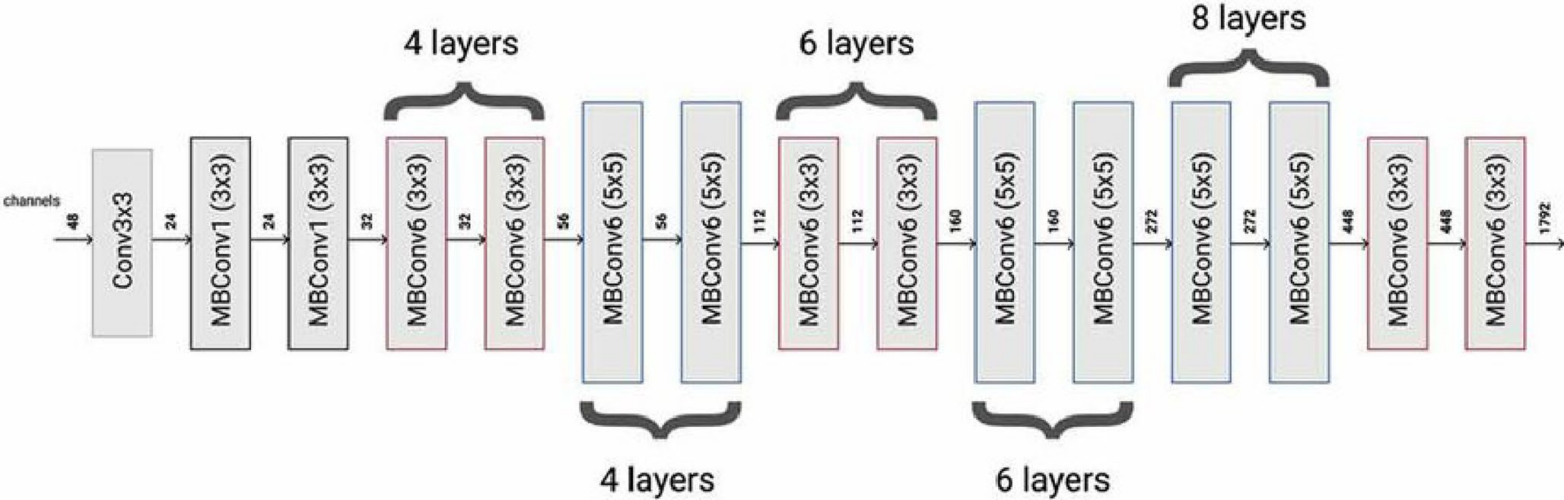
Overview of the EfficientNet-B4 architecture used for feature extraction and classification

These characteristics make EfficientNet‑B4 particularly suitable for our deployment scenario, where inference latency and resource usage are critical constraints. The use of server‑side execution further enables the adoption of this architecture without compromising real‑time performance. The overall model architecture is illustrated in Fig. 5.

The proposed model is both multimodal and multiclass. The multimodal design integrates two distinct sources of information: a visual branch based on EfficientNetB4, which extracts deep image features, and a dense branch that processes structured clinical metadata, including patient age, sex, and lesion location. These branches are merged via a concatenation layer, enabling more accurate predictions by incorporating both visual content and contextual clinical information.

As a multiclass classifier, the system can distinguish among five types of skin lesions: melanoma (MEL), nevus (NV), basal cell carcinoma (BCC), actinic keratosis (AK), and benign keratosis (BKL), thereby facilitating automated differential diagnosis.

To enhance model performance, transfer learning was applied using weights pretrained on ImageNet, followed by progressive fine-tuning. This process involved gradually unfreezing the upper blocks of layers - excluding Batch Normalization layers - to adapt the network more effectively to the dermatological domain.

Optimization was carried out using the AdamW optimizer in combination with the Categorical Crossentropy loss function enhanced by Label Smoothing, which helps mitigate overfitting.

The training process incorporated several callbacks to improve model stability and performance, including EarlyStopping, ModelCheckpoint, ReduceLROnPlateau, TensorBoard, and CSVLogger. Additionally, a custom ProgressiveUnfreezeCallback was implemented to manage the fine-tuning strategy.

The model was trained for 90 epochs and evaluated using stratified k-fold cross-validation (k = 5), with each split divided into stratified training (80%) and validation (20%) sets. Evaluation metrics included Accuracy, Precision, Recall, and F1-Score, computed in both macro and weighted modes to ensure a robust assessment despite class imbalance.

### External and Technical Validation Framework

The technical and external validation of MEL‑IA was designed to address the three scientific questions introduced in the introduction: (i) whether multimodal learning improves classification performance and generalizes to an independent external dataset, (ii) whether the system can be integrated into a hospital‑like environment using interoperability standards, and (iii) whether the end‑to‑end pipeline supports real‑time clinical workflows. To operationalize these questions, the validation framework comprised three components: multimodal performance assessment, interoperability and workflow integration testing, and operational stability analysis under continuous use. These components were complemented by performance benchmarking and a full external validation protocol to evaluate robustness and generalization. A description of all validation procedures is provided in Appendix A.

## Results

### AI Model Classification Performance

**Fig. 6:**
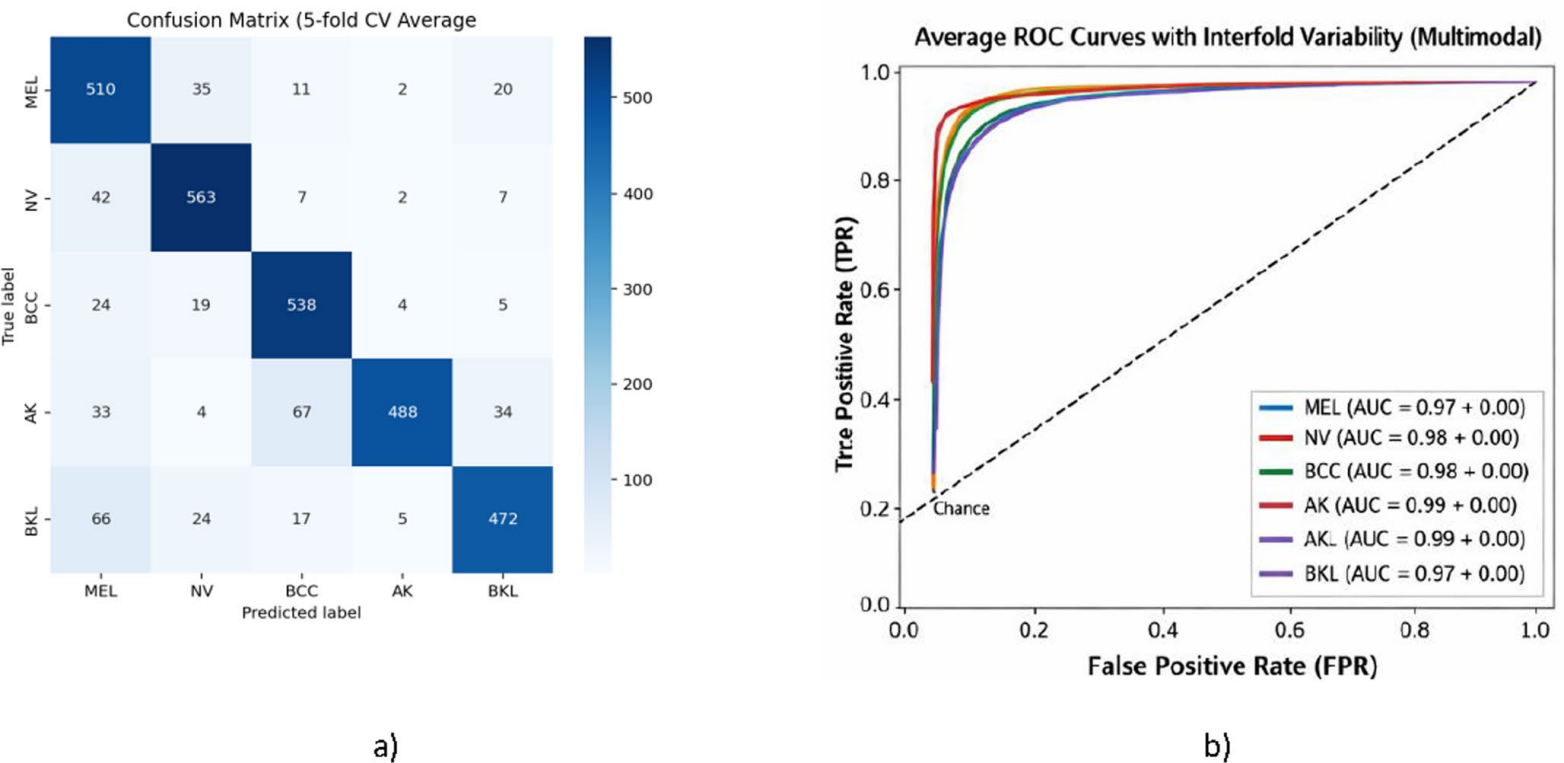
Consolidated performance metrics across five-fold cross-validation: (a) Average confusion matrix summarizing classification accuracy per class; (b) Mean ROC curve with class-wise AUC values averaged over all folds

To evaluate the internal robustness and stability of the model, a five-fold cross-validation was performed using the ISIC 2019 datasets. This procedure allowed us to assess the consistency of the classifier across different data partitions and to quantify inter-fold variability. The consolidated confusion matrix obtained by averaging predictions across folds is shown in Fig. 6a, while Fig. 6b presents the mean ROC curves with class-wise AUC values.

The model demonstrated strong discriminative performance across all diagnostic categories. The highest number of correct predictions was observed for the NV and BCC classes, which consistently achieved high precision, sensitivity, and specificity across folds. Melanoma (MEL) also exhibited robust performance, with an average sensitivity of 0.88, specificity of 0.95, and an F1-score of 0.82, indicating reliable detection of critical malignant lesions.

In contrast, AK and BKL showed greater variability, reflecting the intrinsic heterogeneity of these classes. AK lesions displayed a tendency toward misclassification into BCC or BKL, a pattern consistent with their overlapping keratinization features. Despite this, both classes maintained stable performance across folds, with AK achieving an average balanced accuracy of 0.87 and BKL reaching 0.89.

The ROC analysis further supports the model’s strong discriminative capacity. As shown in Fig. 6b, all classes achieved mean AUC values above 0.97, with minimal inter-fold variability. These results indicate that the model is capable of reliably separating positive and negative cases for each diagnostic category, even in the presence of class imbalance and visual heterogeneity.

Table 4 summarizes the full set of class-wise metrics across folds, including precision, sensitivity, specificity, F1-score, NPV, FNR, FPR, and balanced accuracy, along with the support for each class. For the main performance indicators, 95% confidence intervals were estimated using nonparametric bootstrap resampling with 1,000 iterations, quantifying the variability of the estimates across folds. The model achieved a mean overall accuracy of 0.86, with macro-averaged F1-scores and weighted F1-scores of 0.85. These values remained consistent across folds, underscoring the stability of the classifier and its capacity to generalize reliably within the training domain.

In summary, the cross-validation results demonstrate that the model exhibits strong and consistent performance across all diagnostic categories, with particularly high discriminative power for NV, BCC, and MEL. High AUC values, stable metrics across classes, and limited variability between folds indicate consistent classifier behavior throughout the validation process.

**Table 4:** Class-wise performance metrics across five-fold cross-validation

Class	Precision (PPV)	Recall/ Sensitivity		Spec.	F1-score		NPV	FNR	FPR	AUC	Accuracy	Support
MEL	0.77 (0.73–0.81)	0.88 (0.84–0.91)		0.95 (0.92–0.97)	0.82 (0.78–0.85)		0.98	0.12	0.05	0.97 (0.95–0.98)	0.91 (0.88–0.93)	578
NV	0.88 (0.85–0.91)	0.91 (0.88–0.94)		0.97 (0.95–0.98)	0.89 (0.86–0.92)		0.98	0.09	0.03	0.98 (0.97–0.99)	0.94 (0.92–0.96)	621
BCC	0.89 (0.86–0.92)	0.92 (0.89–0.95)		0.97 (0.95–0.98)	0.90 (0.87–0.93)		0.98	0.08	0.03	0.98 (0.96–0.99)	0.94 (0.92–0.96)	590
AK	0.86 (0.82–0.89)	0.78 (0.73–0.82)		0.95 (0.92–0.97)	0.82 (0.78–0.86)		0.97	0.22	0.04	0.99 (0.98–0.99)	0.87 (0.84–0.90)	626
BKL	0.83 (0.79–0.86)	0.83 (0.79–0.87)		0.96 (0.94–0.98)	0.82 (0.79–0.86)		0.97	0.17	0.04	0.97 (0.95–0.98)	0.89 (0.86–0.91)	584
Global Accuracy			0.86 (0.84–0.88)									
Balanced Accuracy (macro)			0.91 (0.89–0.93)									
Macro F1-score			0.85 (0.82–0.87)									
Weighted F1-score			0.85 (0.83–0.87)									
Macro AUC			0.97 (0.96–0.98)									

#### Qualitative analysis of misclassifications

A qualitative inspection of the misclassifications observed across the five cross-validation folds revealed several consistent patterns that help explain the class-wise performance. Melanoma (MEL) errors were predominantly associated with lesions exhibiting subtle pigmentation gradients, low-contrast borders, or structural patterns that closely resembled benign nevi. These cases typically corresponded to early-stage or clinically ambiguous melanomas, which are known to challenge both automated systems and expert dermatologists. The relatively low false-negative rate (FNR ≈ 0.12) across folds suggests that, despite these difficulties, the model maintained a reliable sensitivity for clinically critical malignant lesions.

Nevus (NV) and basal cell carcinoma (BCC) showed the most stable behavior, with consistently low false-positive and false-negative rates. Misclassifications in these categories were generally linked to atypical presentations, such as heavily pigmented BCCs or nevi with irregular dermoscopic structures. These findings align with the high specificity values observed for both classes (0.97), indicating that the model rarely confuses them with other lesion types.

Actinic keratosis (AK) exhibited the greatest variability in error patterns. Many AK false negatives corresponded to lesions with diffuse erythema or minimal keratinization, which visually overlap with BCC or BKL. Conversely, AK false positives often involved hyperkeratotic lesions with irregular surface patterns, reflecting the inherent heterogeneity of this class and its well-documented clinical similarity to other keratinizing lesions [33, 34]. This explains the comparatively higher FNR (≈ 0.22) and the moderate balanced accuracy observed across folds.

Benign keratosis (BKL) misclassifications were primarily associated with lesions displaying atypical keratin patterns or pigmentation irregularities, which can mimic both melanoma and AK - another phenomenon widely described in dermatological literature [33, 34]. Furthermore, confusion between BCC and AK may stem from overlapping characteristics such as texture, coloration, and anatomical location, particularly in early-stage presentations [35, 36]. Despite these challenges, the model maintained a strong specificity (0.96) and a balanced accuracy close to 0.90, indicating that most BKL cases were correctly identified.

Overall, the qualitative analysis highlights that most misclassifications arise from clinically challenging or visually ambiguous lesions, rather than systematic model failures. These patterns are consistent across folds, reinforcing the stability of the classifier and supporting the quantitative results obtained during cross-validation.

### APP Prototype

The mobile application serves as a user agent, enabling interaction between primary care and specialist physicians. It is compatible with cross-platform solution using the Apache Cordova framework, it supports deployment on both iOS and Android devices and features a responsive design that adapts to various screen sizes.

The underlying information system is supported by a MySQL database, which implements the previously defined data model. Backend services were developed using NodeJS, a JavaScript runtime environment, in conjunction with the Express framework for building web services. Communication between these services and the database is managed via the NodeJS mysql module. All services expose the system’s API and operate over a secure HTTPS channel, ensuring the confidentiality and integrity of processed medical data.

**Fig. 7:**
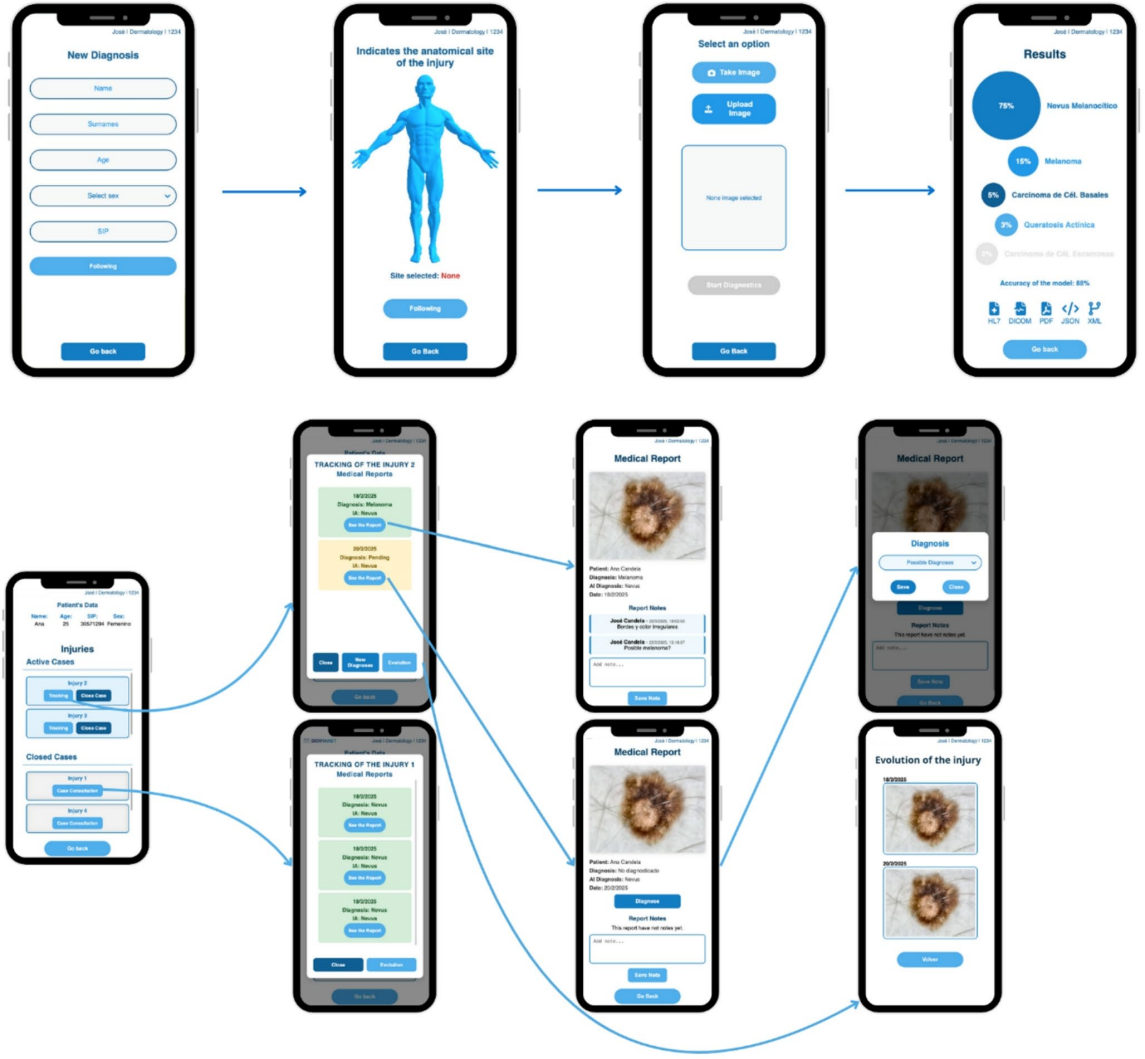
User interface screenshots of the mobile application

The application provides an intuitive interface (see Fig. 7) that facilitates the structured input of clinical data through standardized forms. It also includes an interactive anatomical selector for identifying the affected area. Upon uploading or capturing an image of the skin lesion, the system generates an automated diagnosis, presenting results alongside associated confidence scores. Diagnostic outputs can be exported in multiple standard formats, including HL7, DICOM, PDF, JSON, and XML.

Owing to its compatibility with international standards and the integration channels implemented via the Mirth Connect engine, diagnostic results were successfully integrated into the PACS at the Hospital de San Juan de Alicante. This enabled deployment within a hospital environment and integration with existing hospital infrastructure.

Additionally, the application incorporates dedicated features for clinical follow-up. Healthcare professionals can access both active and closed cases, visualize the temporal evolution of lesions through chronologically organized image sequences, review previous diagnoses, and record new medical observations. The system also supports the updating of existing diagnoses and the closure of clinical episodes, thereby offering a comprehensive tool for longitudinal and well-documented patient management.

### External and Technical Validation Results

The external and technical validation of MEL-IA showed that the system can be integrated within existing clinical infrastructure and that the multimodal model provides measurable performance improvements over the image‑only baseline. System‑level validation confirmed high interoperability with PACS and HIS/RIS, preservation of existing workflows, and stable operation with low error rates and near real‑time processing times. Model‑level evaluation demonstrated that incorporating structured clinical metadata improves classification robustness, particularly for melanoma and basal cell carcinoma, and that the system retains a consistent level of performance across heterogeneous datasets, indicating meaningful generalization capacity.

All external validation materials - including detailed performance tables, class level analyses derived from the independent HAM10000 dataset, interoperability results, operational viability metrics, and the comparison between image model and multimodal models - are provided in Appendix B.

## Discussion

The present study introduces MEL‑IA, a multimodal and interoperable AI system designed to classify skin lesions with high accuracy and function within real hospital workflows. Unlike studies focused mainly on algorithmic performance, MEL‑IA tackles the practical challenges of clinical deployment by combining mobile acquisition, backend inference, and standards based on interoperability (HL7, DICOM, FHIR, Mirth Connect).

### Comparison with State‑of‑the‑Art Models and mHealth Applications

Recent advances in automated skin lesion classification have demonstrated strong performance across multiple deep learning approaches. As summarized in Table 5, models trained on benchmark datasets such as HAM10000 and ISIC typically achieve accuracies between 0.80 and 0.89 and area under the curve (AUC) values ranging from 0.90 to 0.97, depending on the architecture and evaluation protocol [37–40].

**Table 5:** Comparative overview of state-of-the-art methods for skin lesion classification, including model architecture, dataset, evaluation metrics, and key results reported in the literature

Study	Approach	Dataset	Main Metric	Key Results	Conclusion
[37]	Human - AI collaboration	ISIC/dermoscopy	Accuracy/AUC	AUC > 0.90, improved vs. humans alone	Collaboration outperforms either alone
[38]	CNN vs. dermatologists	Dermoscopic images	Accuracy	~ 0.86 (model) vs. ~ 0.79 (dermatologists)	AI comparable or superior to experts
[39]	Feature fusion	HAM10000	Accuracy/AUC	Accuracy ≈ 0.87–0.89	Fusion improves robustness
[40]	CNN ensemble	ISIC	AUC	AUC ≈ 0.90–0.94	Ensembles improve performance
[41]	Vision Transformer (ViT)	ImageNet	Accuracy	State-of-the-art (> 88% top-1)	High capacity, data-intensive
[42]	Swin Transformer	ImageNet/downstream	Accuracy/AUC	Improved performance vs. CNNs	More efficient than standard ViT
[43]	Multimodal	Clinical + image data	Accuracy	+ 2–4% improvement vs. image-only	Clinical data adds value
[44]	Multimodal DL	Dermoscopy + metadata	AUC	AUC ≈ 0.91–0.94	Improved melanoma detection
[45]	Implementation review	—	—	Identifies barriers (interoperability, regulation)	Non-technical challenges dominate
[46]	Systematic review	—	—	Gap between validation and deployment	Limited real-world adoption
[47]	Narrative review	—	—	Low clinical integration	Need for complete systems

Early studies based on CNNs and ensemble strategies reported robust performance, with feature fusion and ensemble methods reaching accuracies of approximately 0.87–0.89 and AUC values above 0.90 [39, 40]. In addition, human - AI collaboration frameworks have demonstrated improvements over standalone human performance, achieving AUC values exceeding 0.90 [37], while some models have been shown to outperform dermatologists in controlled classification tasks (e.g., ≈ 0.86 vs. ≈ 0.79 accuracy) [38].

More recently, architectures based on transformer such as Vision Transformers (ViT) [41] and Swin Transformer [42] have achieved competitive results in general computer vision tasks due to their ability to model global contextual information. However, their application in dermatology remains limited, mainly due to their high computational cost and the lack of validation in real clinical environments.

Multimodal approaches that integrate clinical metadata with dermoscopic images have further improved classification performance, typically providing gains of 2–4% in accuracy or AUC, particularly in challenging classes such as melanoma and basal cell carcinoma [43, 44]. Despite these encouraging results, most studies rely on internal validation using curated datasets. As highlighted in recent reviews, a significant gap persists between high experimental performance and deployment in real clinical environments, mainly due to challenges related to interoperability, workflow integration, and regulatory constraints [45–47].

On the other hand, mobile applications oriented to consumers such as SkinVision[48], Miiskin[49], Aysa[50], and DermaScan[51] represent a different category: they function as self-assessment tools without clinical governance, DICOM generation, HL7/FHIR support, or connectivity with PACS or HIS/RIS systems. Their unimodal design and lack of traceability further limit their suitability for regulated clinical workflows.

Within this broader landscape, MEL-IA achieves competitive internal performance (accuracy = 0.86; macro-AUC = 0.97) while providing capabilities rarely combined in prior work: mobile dermatoscopic acquisition, multimodal inference, PACS/EHR interoperability, and external validation. Although its macro-F1 score decreases under external dataset evaluation (0.67 on HAM10000), the system maintains strong performance in NV and BCC and demonstrates great generalization capacity beyond the training distribution which has been identified as essential for clinical translation [52].

Beyond comparative performance, these results illustrate the practical implications of integrating the system within hospital environments. By combining dermatoscopic images with structured metadata and generating standardized DICOM and HL7/FHIR outputs, MEL-IA supports real dermatology workflows rather than functioning as a standalone image analysis tool. This multimodal and interoperable design enables triage support, documentation, and longitudinal follow-up within PACS/EHR systems, aligning the system with practical clinical use rather than consumer self-assessment applications.

### Scientific Interpretation of Results and Generalization Capacity

Cross‑validation shows strong, consistent performance across lesion categories, with particularly high discriminative power for NV and BCC. MEL achieved sensitivity 0.88 and specificity 0.95, ensuring reliable detection of malignant lesions. AK and BKL displayed more variability, consistent with their heterogeneity.

External validation on HAM10000 provided a more stringent assessment of generalization. Given the differences in acquisition devices, class imbalance, and domain shift, MEL-IA showed a reasonable level of robustness. Melanoma reached a sensitivity of 0.60 and a specificity of 0.98 (FNR 0.40), reflecting both its discriminative capacity and the known difficulty of detecting subtle lesions in external datasets. NV and BCC also maintained high sensitivity (0.94 and 0.72) and specificity (0.82 and 0.98), while AK showed the lowest sensitivity (0.27) but very high specificity (0.93). Overall accuracy reached 0.84, indicating that the model preserved meaningful performance despite the heterogeneity of the external dataset.

Qualitative analysis revealed clinically plausible error patterns. Melanoma false negatives typically involved subtle pigmentation or blurred borders, while AK errors reflected high intra‑class variability and overlap with BKL/BCC. Overall, errors mainly arose from visually ambiguous cases rather than systematic faults. These results confirm that MEL‑IA generalizes reliably under heterogeneous conditions.

### Clinical Implications and Ethical Considerations

Integrating MEL-IA within a hospital setting offers several operational benefits, including rapid image acquisition, automated analysis, and complete integration with PACS and EHR systems. This may reduce manual workload and diagnostic delays, particularly in primary care settings where non-specialists require decision support for effective triage.

However, the presence of false negatives - especially in melanoma detection - highlights the need for continued clinician oversight. In this context, MEL-IA is designed as a decision-support tool rather than an autonomous diagnostic system, aligning with key ethical principles such as transparency, accountability, and clinician oversight.

The system also enables a workflow that connects mobile acquisition with hospital systems, supporting teledermatology and facilitating remote assessment while improving access to care in underserved or resource limited areas. The use of standardized data formats ensures traceability and supports longitudinal patient follow-up.

From an ethical and regulatory perspective, MEL-IA prioritizes data privacy and security by ensuring that all patient data are stored within hospital-controlled infrastructures. Additionally, the generation of traceable HL7/FHIR messages and DICOM objects provides a structured and auditable data flow, in contrast to many consumer-grade applications that rely on unregulated cloud storage.

### Feasibility and Challenges of Integration with Hospital Systems

The successful deployment of MEL-IA provides evidence of the feasibility of integrating AI systems into routine clinical workflows. The use of Mirth Connect as an interoperability engine enabled communication with PACS, HIS/RIS, and EHR systems. The platform generates DICOM-compliant objects with complete metadata, transmits them to PACS, and exchanges clinical information using standardized HL7/FHIR messaging.

Beyond technical integration, the system was operationally validated in a real clinical environment, processing 980 dermatology studies with over 99% uptime, sub-second median latency, and more than 99% successful PACS ingestion. These results indicate that MEL-IA can operate within routine hospital workflows.

Despite this successful deployment, several challenges remain. Integration across heterogeneous hospital infrastructures requires flexible configuration of interoperability channels and routing rules. In addition, cybersecurity, system uptime, and continuous AI model maintenance demand close collaboration between clinical, technical, and IT teams.

Furthermore, variability in PACS, HIS/RIS, and EHR configurations, along with strict data governance and privacy requirements, may limit the portability of such systems across institutions. These constraints highlight the importance of robust engineering practices, standardization, and multidisciplinary coordination to ensure safe, scalable, and sustainable integration of AI solutions into healthcare environments.

### Limitations and Future Work

Although the app supports mobile image capture, current evaluation relied solely on public dermatoscopic datasets; real smartphone testing with compatible dermatoscopic attachments is necessary for practical validation. The datasets also lack dermatologist reports and multi-expert annotations, preventing direct expert comparison. Incorporating curated expert-labeled data and reader studies will strengthen clinical validation.

Finally, despite the successful technical integration, MEL-IA’s clinical impact must be assessed prospectively. Future work will include clinician-in-the-loop trials, usability evaluations, workflow analyses, and the incorporation of imbalance-handling strategies to extend the model to underrepresented lesion types such as DF, VASC, and SCC. These steps will be essential to determine effectiveness, safety, and scalability in routine practice.

Additional limitations include the absence of calibrated clinical thresholds, potential class imbalance effects - particularly for melanoma and AK - and the influence of acquisition variability across institutions. While operational validation demonstrates feasibility, formal prospective studies are required to quantify diagnostic impact, workflow efficiency, and patient‑level outcomes.

## Conclusions

In this work, we presented MEL-IA, a multimodal and interoperable AI system for skin lesion classification in real time fully integrated into hospital workflows. By combining dermatoscopic image analysis with structured clinical metadata and operating within an HL7/DICOM/FHIR-based architecture via Mirth Connect, the system functions as an embedded component of the clinical information ecosystem rather than a standalone algorithm.

The model showed robust internal performance (accuracy ≈ 0.86; macro AUC ≈ 0.97), with particularly strong results for NV and BCC. External validation on HAM10000 confirmed its generalization capacity, maintaining high sensitivity and specificity across most classes despite heterogeneous acquisition conditions. Misclassifications were mainly concentrated in clinically ambiguous lesions, such as early melanomas or heterogeneous keratinizing lesions, reinforcing the need for clinician oversight and positioning MEL-IA as a decision-support tool rather than an autonomous diagnostic system.

An important aspect of this work is the implementation of interoperability mechanisms that support the integration of AI tools into hospital environments. MEL-IA integrates successfully with the PACS and HIS/RIS of Hospital de San Juan de Alicante, generating DICOM-compliant outputs and HL7/FHIR messages that feed directly into the patient’s EHR, ensuring traceability, auditability, and alignment with clinical governance requirements.

In summary, the results support the technical feasibility, interoperability, and operational viability of MEL IA within hospital environments. By unifying multimodal AI-based diagnosis with interoperability designed for clinical environments, it provides a framework for integrating dermatological decision support into existing clinical workflows. Future work will focus on prospective clinical evaluation, smartphone image acquisition, and expanding lesion categories to enhance applicability and safety in real clinical environments.

## Supplementary Information

Below is the link to the electronic supplementary material.

Supplementary Material 1

Supplementary Material 2

## Data Availability

No datasets were generated or analysed during the current study.

## Footnotes

1 Mirth Connect: https://www.nextgen.com/solutions/interoperability/mirth-integration-engine.
