# Supplementary material for: MEL-IA: An Interoperable AI System for Multimodal Skin Lesion Classification in Hospital Settings
Source: J Med Syst. 2026 Jun 12;50(1):95. doi: 10.1007/s10916-026-02424-y (PMC13260036; doi:10.1007/s10916-026-02424-y)
Supplement: Supplementary file 1 — Supplementary Material 1 [file 10916_2026_2424_MOESM1_ESM.docx]

**Appendix A. External and Technical Validation Framework**

**A.1 External Validation Protocol**

The external validation strategy combined internal and external evaluation. Internal validation used stratified five‑fold cross‑validation on the ISIC 2019 datasets (BCN_20000 and MSK datasets) to ensure proportional class representation and assess model stability across data partitions. External validation was performed on the HAM10000 dataset (n = 9701), which was entirely unseen during training and provided an independent assessment under heterogeneous acquisition conditions.

From the resulting confusion matrices, clinically relevant metrics were derived, including sensitivity, specificity, predictive values, false‑negative and false‑positive rates, and balanced accuracy. A qualitative analysis of misclassified cases identified recurring error patterns, such as subtle melanomas with low‑contrast borders or the high morphological variability of actinic keratoses. Confidence intervals (95%) were computed using 1,000‑sample bootstrapping. P‑values were not calculated because ISIC 2019 and HAM10000 lack multiple independent annotations or paired dermatologist assessments, preventing statistically valid hypothesis testing. All analyses were performed using Python (NumPy, SciPy, scikit‑learn).

**A.2 Technical Performance Evaluation**

The first component of the validation framework assessed whether incorporating structured clinical metadata improves classification performance beyond image‑only learning. Two models were trained under identical conditions: an EfficientNet‑B4 model using dermatoscopic images alone (unimodal), and a multimodal variant integrating images and metadata. Both were evaluated on the same independent test set using AUC‑ROC, precision, specificity, and F1‑score. The hypothesis was considered supported if the multimodal model matched or exceeded the image‑only baseline, particularly in AUC and sensitivity, which are the most clinically relevant metrics for early melanoma detection.

**A.3 Interoperability and Workflow Integration Evaluation**

A second hypothesis examined whether MEL‑IA could be integrated into a hospital‑like environment without altering existing clinical workflows. The system was deployed in an infrastructure replicating real hospital conditions, including PACS, HIS/RIS, and an institutional integration engine. MEL‑IA was required to generate DICOM objects enriched with complete metadata, transmit them reliably to PACS, and exchange administrative and clinical information through HL7/FHIR messages. Successful integration was defined by the absence of transmission errors, full compatibility with existing systems, and seamless operation from the clinician’s perspective. Quantitative indicators included the proportion of successfully integrated studies, transmission or conversion error rates, and the time elapsed from image acquisition to PACS availability. Representative workflow diagrams and HL7/DICOM messages were documented to demonstrate standards compliance.

**A.4 Operational Viability Assessment**

The third hypothesis evaluated MEL‑IA’s stability and reliability as a continuously running clinical system. Metrics included the total number of processed studies, the percentage completed without errors, end‑to‑end processing time (from image capture to PACS storage), and system uptime based on incident and network logs. Special attention was given to architectural modularity, particularly the ability to update or replace the AI model without disrupting interoperability layers such as DICOM or HL7/FHIR. The hypothesis was supported if the system demonstrated sustained operation, low error rates, and the capacity to integrate updates without workflow interruptions.

**A.5 Performance Benchmarking and Deployment Strategy**

To assess computational feasibility under realistic deployment conditions, inference latency, complete pipeline time, and memory usage were benchmarked in the production server environment used for clinical integration. Measurements included (1) model loading, (2) preprocessing of incoming images and metadata, (3) multimodal inference, and (4) generation of the final structured output. Pipeline latency was defined as the total time from image capture to the return of the diagnostic result. Inference was executed entirely on a backend server, allowing the mobile application to focus solely on image capture and metadata submission and enabling the use of more accurate models such as EfficientNet‑B4 without compromising real‑time performance.
