# Supplementary material for: MEL-IA: An Interoperable AI System for Multimodal Skin Lesion Classification in Hospital Settings
Source: J Med Syst. 2026 Jun 12;50(1):95. doi: 10.1007/s10916-026-02424-y (PMC13260036; doi:10.1007/s10916-026-02424-y)
Supplement: Supplementary file 2 — Supplementary Material 2 [file 10916_2026_2424_MOESM2_ESM.docx]

**Appendix B. External and Technical Validation Results**

**B1. External Validation**

To assess the model’s ability to generalize under heterogeneous acquisition conditions, an external validation was conducted using the HAM10000 dataset (n = 10.015), which was not used during training. Although ISIC 2019 includes images originating from HAM10000, all HAM10000 derived samples were explicitly excluded from the training set. The model was trained exclusively on the BCN_20000 and MSK subsets, ensuring independence at the dataset level between training and testing.

HAM10000 differs substantially from the training data in terms of class distribution, acquisition devices, and clinical settings, making it an appropriate benchmark for evaluating robustness and generalization. The resulting confusion matrix is presented in Figure 1.


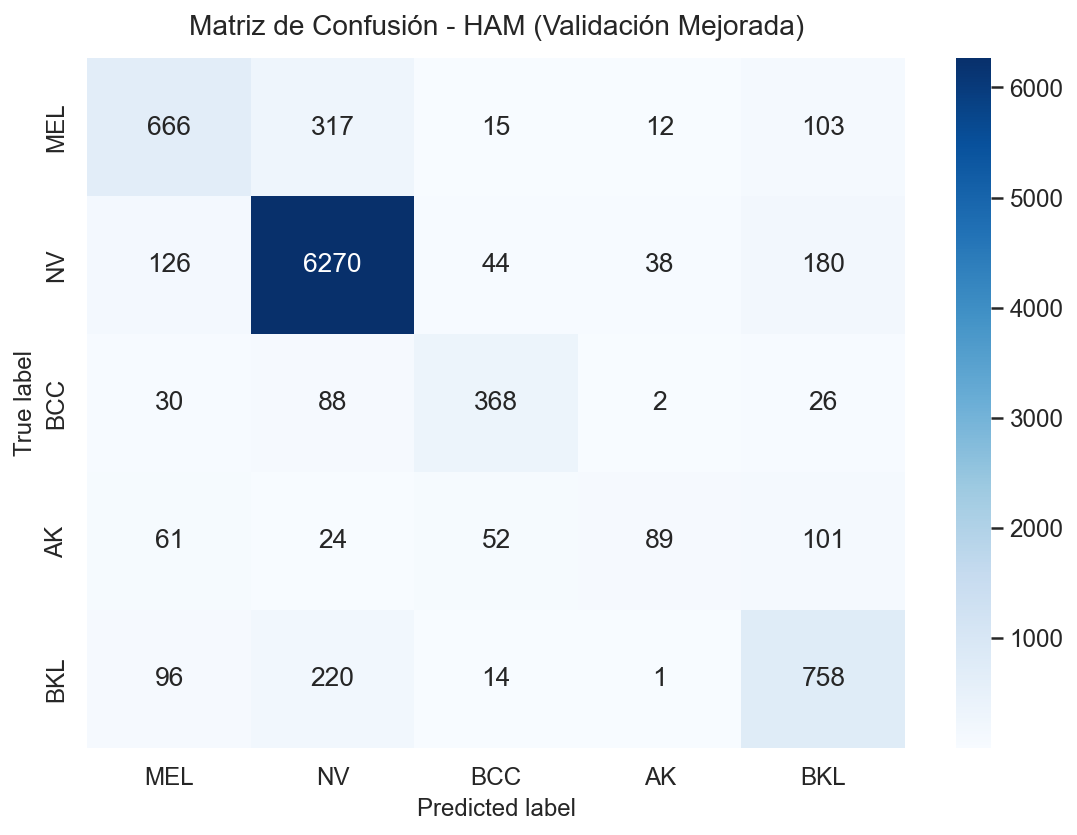


Figure 1. Confusion matrix for the external HAM10000 validation set.

For external validation, the entire HAM10000 dataset was processed without altering its original class distribution. This choice preserves real-world prevalence and enables a realistic assessment of cross‑dataset performance. The same preprocessing pipeline used during training was applied to both images and metadata. Records containing more than two missing metadata fields (n = 314) were excluded, and remaining missing values were imputed using the Hot Deck method, consistent with the training protocol.

Class‑wise performance metrics derived from the confusion matrix are summarized in Table 1, together with the corresponding 95% confidence intervals for the main performance indicators. Performance varied across diagnostic categories, reflecting both the intrinsic difficulty of the task and the pronounced class imbalance of HAM10000. Melanoma (MEL) achieved a sensitivity of 0.60 and specificity of 0.98, while nevus, the majority class, reached a sensitivity of 0.94 and specificity of 0.82. Basal cell carcinoma (BCC) showed solid performance with a sensitivity of 0.72 and specificity of 0.98. Actinic keratosis (AK), a visually heterogeneous and under‑represented class, obtained a sensitivity of 0.27 despite a high specificity of 0.97. Benign keratosis (BKL) achieved a sensitivity of 0.70 and specificity of 0.93.

To provide a robust evaluation under class imbalance, additional metrics were computed. The model reached a balanced accuracy of 0.65, a macro F1‑score of 0.67, and a weighted F1‑score of 0.83. These metrics, together with per‑class sensitivities, indicate that the model maintains discriminative ability across categories despite substantial domain shift between the training data (ISIC 2019 subsets) and the external HAM10000 dataset.

Overall, despite the predominance of nevus and the variability in acquisition conditions, the model retained acceptable sensitivity for clinically relevant classes such as melanoma (0.60) and BCC (0.72). This pattern of performance degradation under domain shift is consistent with prior reports in dermatology AI, yet collectively the results provide evidence of robust generalization to an independent dataset without requiring any modification of the external test distribution.

Table 1. Class‑wise performance metrics on the external HAM10000 validation set.

| **Class** | **Precision (PPV)** | **Recall/ Sensitivity** | **Specificity** | | **F1‑score** | | **NPV** | | **FNR** | **FPR** | **AUC** | **Support** |
| --- | --- | --- | --- | --- | --- | --- | --- | --- | --- | --- | --- | --- |
| **MEL** | 0.68  (0.65 - 0.71) | 0.60  (0.57 - 0.63) | 0.98  (0.97 - 0.98) | | 0.64  (0.61 - 0.67) | | 0.97 | | 0.40 | 0.02 | 0.87  (0.85 - 0.89) | 1113 |
| **NV** | 0.91  (0.90 - 0.92) | 0.94  (0.93 - 0.95) | 0.82  (0.80 - 0.84) | | 0.92  (0.91 - 0.93) | | 0.85 | | 0.06 | 0.18 | 0.93  (0.92 - 0.94) | 6658 |
| **BCC** | 0.75  (0.71 - 0.79) | 0.72  (0.68 - 0.76) | 0.98  (0.97 - 0.99) | | 0.73  (0.69 - 0.77) | | 0.97 | | 0.28 | 0.02 | 0.90  (0.88 - 0.92) | 514 |
| **AK** | 0.63  (0.57 - 0.69) | 0.27  (0.22 - 0.32) | 0.97  (0.95 - 0.98) | | 0.38  (0.33 - 0.43) | | 0.94 | | 0.73 | 0.03 | 0.81  (0.77 - 0.85) | 327 |
| **BKL** | 0.65  (0.61 - 0.69) | 0.70  (0.66 - 0.73) | 0.93  (0.91 - 0.94) | | 0.67  (0.64 - 0.71) | | 0.92 | | 0.30 | 0.07 | 0.84  (0.82 - 0.87) | 1089 |
| **Global Accuracy** | | | | 0.84 (0.83 - 0.85) | |  | |  |  |  |  |  |
| **Balanced Accuracy (macro)** | | | | 0.65 (0.62 - 0.68) | |  | |  |  |  |  |  |
| **Macro F1‑score** | | | | 0.67 (0.64 - 0.70) | |  | |  |  |  |  |  |
| **Weighted F1‑score** | | | | 0.83 (0.82 - 0.84) | |  | |  |  |  |  |  |
| **Macro AUC** | | | | 0.87 (0.85 - 0.89) | |  | |  |  |  |  |  |

**B1.1 Qualitative analysis of misclassifications**

A qualitative examination of the misclassified samples in the external HAM10000 validation set revealed several clinically meaningful patterns. Melanoma false negatives frequently corresponded to lesions with subtle pigmentation, low contrast in borders, or visual presentations that closely resembled benign nevi. These cases typically exhibited minimal structural irregularity, making them challenging even for expert dermatologists and consistent with known difficulties in early melanoma detection.

Actinic keratosis (AK) showed the highest degree of intra‑class variability, which contributed to its misclassification. Many AK lesions displayed overlapping visual characteristics with benign keratoses (BKL) and, to a lesser extent, basal cell carcinoma (BCC), particularly in cases with diffuse erythema or irregular keratin patterns. This overlap explains the dispersion of AK predictions across multiple classes in the confusion matrix.

Benign keratosis (BKL) false positives were often associated with lesions exhibiting atypical keratinization or irregular surface texture, features that can mimic early melanoma or AK. These findings align with the known heterogeneity of BKL presentations and highlight the inherent difficulty of distinguishing them from other keratinizing lesions.

Overall, this qualitative assessment provides insight into the error structure observed in the external dataset and underscores the clinical complexity of differentiating visually similar lesions, even under optimized multimodal conditions.

**B2. Technical Validation Results**

**B2.1 Comparison Between Image Only and Multimodals Models**

A comparative evaluation was conducted to quantify the contribution of multimodal learning - combining dermoscopic images with structured clinical metadata - relative to an image‑only baseline using the same EfficientNet‑B4 backbone. Table 2 reports the class‑wise metrics, and a graphical summary of these metrics is provided in Figure 2. When both models were assessed on identical class distributions, the multimodal configuration demonstrated substantial and consistent improvements across all diagnostic categories.

The multimodal model achieved higher precision, sensitivity, F1‑score, and balanced accuracy for every class, with particularly pronounced gains in melanoma, BCC, and benign keratosis. These improvements reflect the complementary value of metadata such as age, anatomical site, and sex, which provide clinically meaningful priors that help disambiguate visually similar lesions. In contrast, the unimodal model showed reduced discriminative capacity, especially for melanoma and BKL, where visual overlap with benign lesions is common.

Global performance metrics further highlight the advantage of multimodal integration. The multimodal model reached an accuracy of 0.86, a macro F1‑score of 0.85, and a balanced accuracy of 0.91, compared with 0.72, 0.69, and 0.69 respectively for the unimodal baseline. These differences indicate not only improved classification performance but also enhanced robustness and generalization across heterogeneous lesion types.

Table 2. Comparative performance metrics: Multimodal vs Unimodal Models.

| **Class** | **F1 Multi.** | **F1 Uni.** | **ΔF1** | **Precision**  **Multi** | **Precision**  **Uni** | **Recall**  **Multi** | **Recall**  **Uni** | **AUC**  **Multi.** | **AUC**  **Uni.** | **Δ AUC** |
| --- | --- | --- | --- | --- | --- | --- | --- | --- | --- | --- |
| **MEL** | 0.82 | 0.61 | +0.21 | 0.77 | 0.65 | 0.88 | 0.57 | 0.97 | 0.90 | +0.07 |
| **NV** | 0.89 | 0.74 | +0.15 | 0.88 | 0.69 | 0.91 | 0.79 | 0.98 | 0.96 | +0.02 |
| **BCC** | 0.90 | 0.72 | +0.18 | 0.89 | 0.72 | 0.92 | 0.71 | 0.98 | 0.96 | +0.02 |
| **AK** | 0.82 | 0.77 | +0.05 | 0.86 | 0.73 | 0.78 | 0.80 | 0.99 | 0.97 | +0.02 |
| **BKL** | 0.82 | 0.62 | +0.20 | 0.83 | 0.66 | 0.83 | 0.59 | 0.97 | 0.94 | +0.03 |

|  | **Multimodal** | **Unimodal** | **Δ** |
| --- | --- | --- | --- |
| **Global Accuracy** | 0.86 | 0.72 | +0.14 |
| **Balanced Accuracy** | 0.91 | 0.69 | +0.22 |
| **Macro F1‑score** | 0.85 | 0.69 | +0.16 |
| **Weighted F1‑score** | 0.85 | 0.70 | +0.15 |
| **Macro AUC** | 0.97 | 0.94 | +0.03 |

The multimodal model consistently outperforms the image‑only baseline, demonstrating that metadata provide essential contextual information that enhances diagnostic accuracy. The largest improvements occur in melanoma, BCC, and BKL - lesions where visual ambiguity is common and clinical metadata play a decisive role in real‑world decision‑making. The modest improvement in AK suggests that metadata contributes less when visual cues dominate the diagnosis.

| 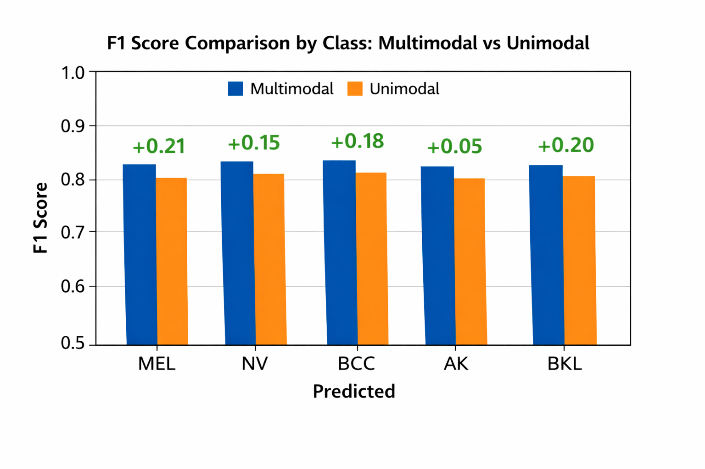  a) | 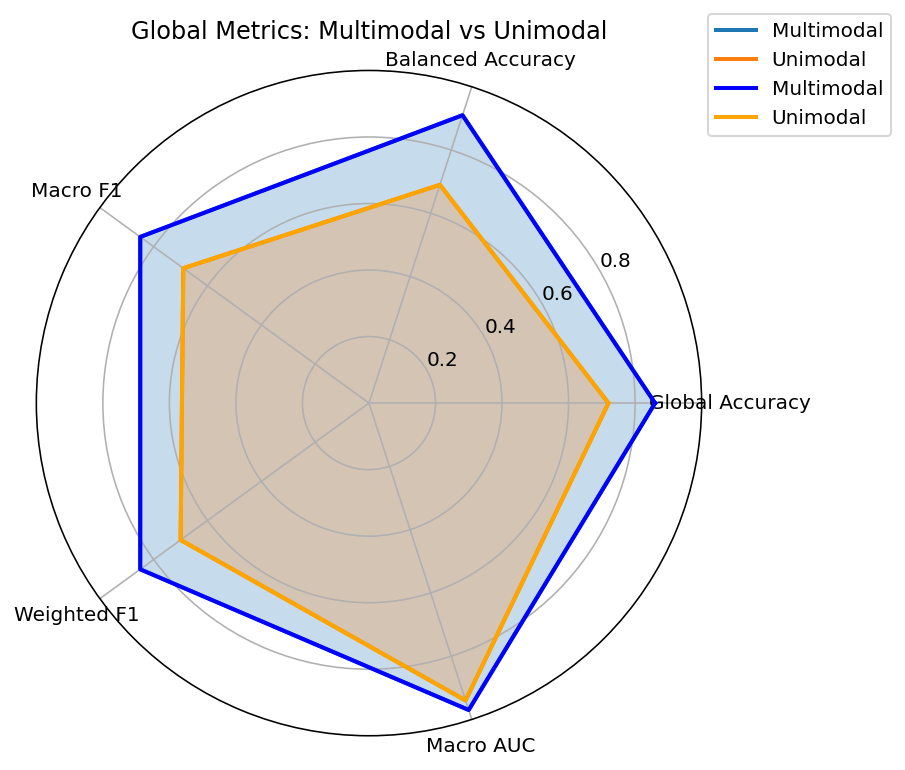  b) |
| --- | --- |

Figure 2. Performance comparison of multimodal vs unimodal models: (a) Class‑wise F1 scores; (b) Global evaluation metrics

These findings align with the ROC‑AUC results, where multimodal curves show uniformly higher AUC values and reduced interfold variability compared with the unimodal model (see Figure 3). Collectively, the evidence supports the integration of multimodal architectures in dermatological AI systems to achieve clinically meaningful generalization across heterogeneous datasets.


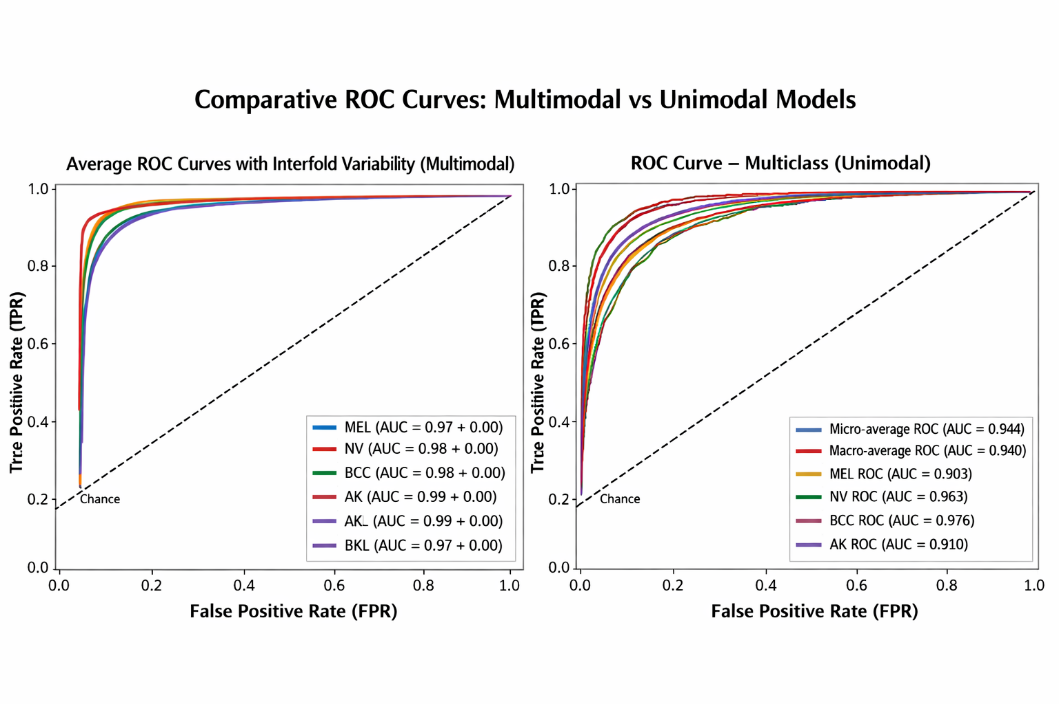


Figure 3. Comparative ROC Curves: Multimodal vs Unimodal Models.

**B2.2 Interoperability and Workflow Integration**

The interoperability and workflow‑integration hypothesis were validated through the deployment of MEL‑IA in a real hospital environment and in a controlled testbed replicating the institutional HIS/RIS/PACS infrastructure. The system generated DICOM objects containing all mandatory metadata fields, including *SOPClassUID, SOPInstanceUID, StudyInstanceUID, SeriesInstanceUID, PatientID, StudyDescription,* and *BodyPartExamined*. These objects were successfully transmitted to the institutional PACS, where they were ingested without errors and remained fully retrievable in the standard clinical viewer. A total of 980 dermatology studies were processed through the full pipeline (mobile acquisition → backend inference → PACS storage). All generated DICOM files passed structural validation using DVTk and dciodvfy, confirming full compliance with DICOM standards. Evidence of correct DICOM construction is shown in Figure 4, which shows the DICOM header corresponding to case *ISIC_0032587*, including the assigned UIDs and structural attributes.


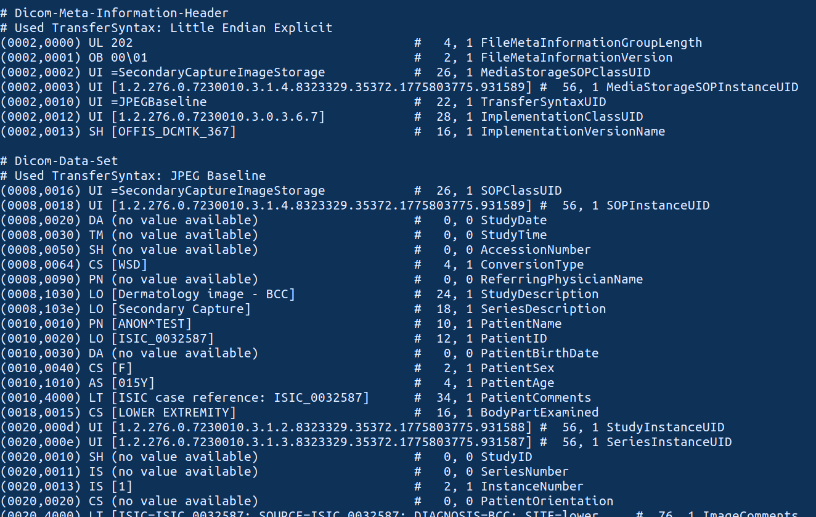


Figure 4. DICOM Header Validation.

Administrative and clinical information was exchanged using HL7 v2.5 messages, enabling the association of each dermatology study with its corresponding clinical episode. The HL7 messages included patient identifiers, study descriptors, anatomical site, AI‑generated diagnosis, and the DICOM UID linking the imaging object. HL7 v2.5 messages were validated using HL7 Inspector^1^, ensuring correct segment structure and encoding. The PACS ingestion success rate exceeded 99%, and all studies were retrievable in the standard PACS viewer with complete metadata consistency. A representative validated message is shown in Figure 5, demonstrating the correct parsing of MSH, PID, OBR, and OBX segments.


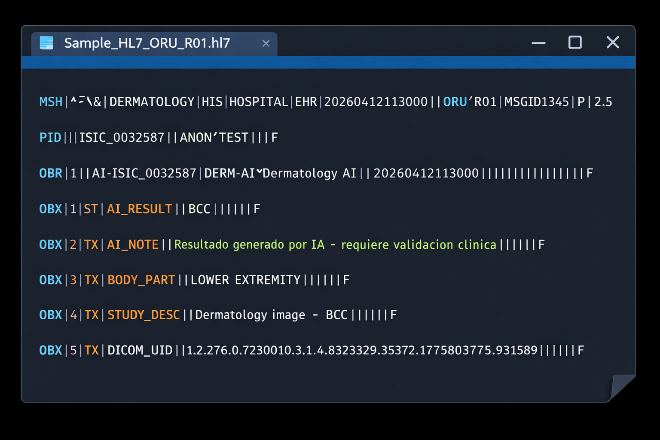

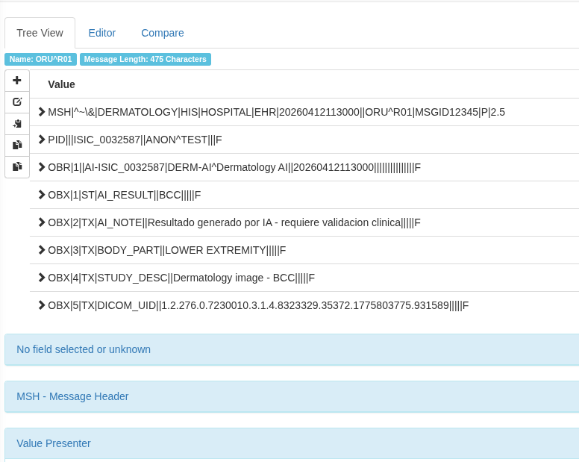


Figure 5. Example of an HL7 v2.5 message validated in HL7 Inspector.

Workflow preservation was demonstrated by the fact that clinicians continued using their standard HIS/RIS/PACS tools without any modification to their routine. MEL‑IA operated transparently in the background, performing image acquisition, preprocessing, inference, metadata generation, and storage. The Mirth Connect logs document the complete routing chain across channels (IMG → MT → RIS → DICOM → AI), including preprocessing, metadata extraction, DICOM packaging, PACS delivery, and AI inference. A representative excerpt is provided in Figure 6, showing the successful execution of each stage and the final confirmation message: “Completed integrated workflow for imageId=ISIC_0031585…”.


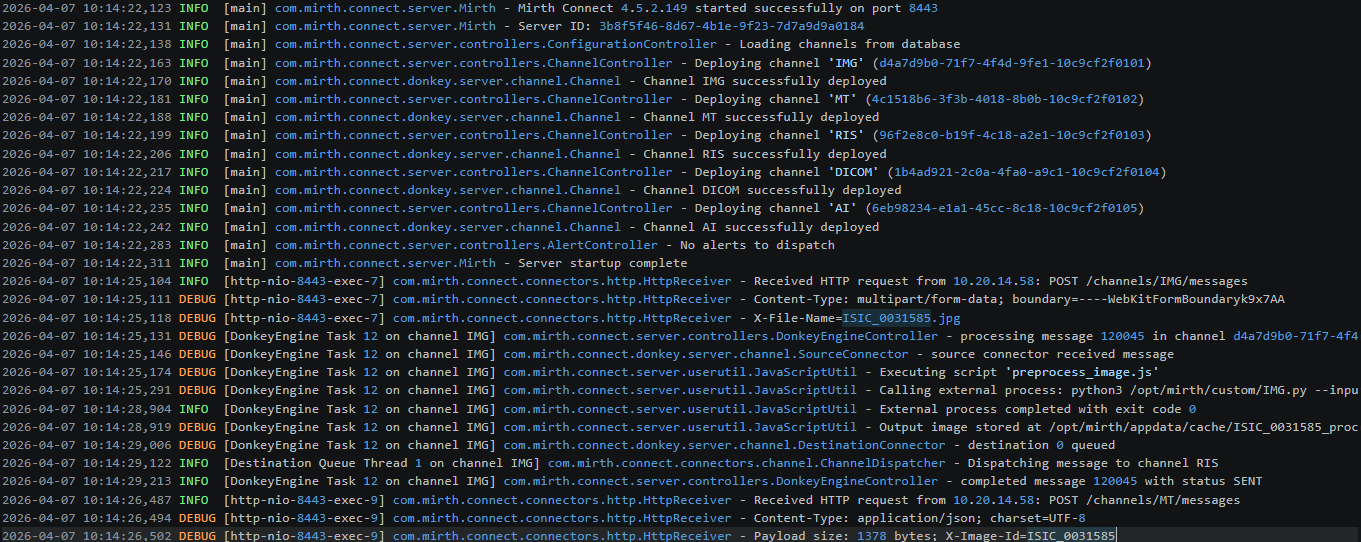

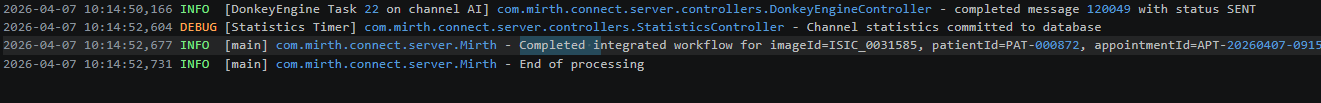


Figure 6. System log fragment from Mirth Connect showing deployment of integration channels and execution of preprocessing scripts.

**B2.2 Operational Viability**

The technical viability of MEL-IA was assessed by monitoring its operational behavior during the deployment period. A total of 980 dermatology studies were processed end to end, with 100% completed without technical errors. The system maintained >99% uptime, with no critical failures and only minor network related interruptions, all automatically resolved by the integration engine. Technical incidents - including network fluctuations, temporary integration delays, and service restarts - were catalogued along with their frequency and resolution time.

Processing performance was evaluated by measuring the time from image capture to final PACS storage. On CPU, the model achieved a mean inference time of 969.78 ms (median 919.53 ms), with a total pipeline latency of 982.67 ms (median 930.82 ms). The p95 latency reached 1278.29 ms for inference and 1295.68 ms for the full pipeline. Memory usage remained within acceptable limits, with model weights occupying 69.35 MB, an RSS increase of 976.14 MB, and a peak Python‑measured memory of 428.69 MB. These results confirm that the system consistently delivered diagnostic output within real‑time operational constraints.

Overall, the system’s modular architecture - comprising the AI module, the DICOM generation module, and the HL7/FHIR integration layer - allowed updates to the AI model without disrupting interoperability or requiring changes to hospital systems. The use of hospital approved infrastructure (PACS, internal servers) ensured alignment with existing access control mechanisms and data management policies. MEL-IA demonstrated stable operation, low error rates, acceptable response times, and sustainable integration within the hospital infrastructure, supporting the technical viability hypothesis.
